# On Predicting lung cancer subtypes using ‘omic’ data from tumor and tumor-adjacent histologically-normal tissue

**DOI:** 10.1186/s12885-016-2223-3

**Published:** 2016-03-04

**Authors:** Arturo López Pineda, Henry Ato Ogoe, Jeya Balaji Balasubramanian, Claudia Rangel Escareño, Shyam Visweswaran, James Gordon Herman, Vanathi Gopalakrishnan

**Affiliations:** Department of Biomedical Informatics, University of Pittsburgh School of Medicine, 5607 Baum Boulevard, 15206 Pittsburgh, PA USA; Department of Computational Genomics, National Institute of Genomic Medicine, Periferico Sur No. 4809, Col. Arenal Tepepan, Tlalpan, 14610 Mexico City Mexico; Division of Hematology/Oncology, Department of Medicine, University of Pittsburgh School of Medicine, UPMC Cancer Pavilion, 5150 Centre Avenue, 15232 Pittsburgh, PA USA

**Keywords:** Bayes Theorem, Adenocarcinoma of Lung, Squamous Cell Carcinoma, DNA Methylation

## Abstract

**Background:**

Adenocarcinoma (ADC) and squamous cell carcinoma (SCC) are the most prevalent histological types among lung cancers. Distinguishing between these subtypes is critically important because they have different implications for prognosis and treatment. Normally, histopathological analyses are used to distinguish between the two, where the tissue samples are collected based on small endoscopic samples or needle aspirations. However, the lack of cell architecture in these small tissue samples hampers the process of distinguishing between the two subtypes.

Molecular profiling can also be used to discriminate between the two lung cancer subtypes, on condition that the biopsy is composed of at least 50 % of tumor cells. However, for some cases, the tissue composition of a biopsy might be a mix of tumor and tumor-adjacent histologically normal tissue (TAHN). When this happens, a new biopsy is required, with associated cost, risks and discomfort to the patient. To avoid this problem, we hypothesize that a computational method can distinguish between lung cancer subtypes given tumor and TAHN tissue.

**Methods:**

Using publicly available datasets for gene expression and DNA methylation, we applied four classification tasks, depending on the possible combinations of tumor and TAHN tissue. First, we used a feature selector (ReliefF/Limma) to select relevant variables, which were then used to build a simple naïve Bayes classification model. Then, we evaluated the classification performance of our models by measuring the area under the receiver operating characteristic curve (AUC). Finally, we analyzed the relevance of the selected genes using hierarchical clustering and IPA® software for gene functional analysis.

**Results:**

All Bayesian models achieved high classification performance (AUC > 0.94), which were confirmed by hierarchical cluster analysis. From the genes selected, 25 (93 %) were found to be related to cancer (19 were associated with ADC or SCC), confirming the biological relevance of our method.

**Conclusions:**

The results from this study confirm that computational methods using tumor and TAHN tissue can serve as a prognostic tool for lung cancer subtype classification. Our study complements results from other studies where TAHN tissue has been used as prognostic tool for prostate cancer. The clinical implications of this finding could greatly benefit lung cancer patients.

**Electronic supplementary material:**

The online version of this article (doi:10.1186/s12885-016-2223-3) contains supplementary material, which is available to authorized users.

## Background

Lung cancer is the leading cause of human cancer death in both sexes in the United States. In 2014, there was an estimate of 224,210 new cases, while 159,260 patients were estimated to have died from the disease [1]. Cigarette smoking is the main risk factor for the development of lung cancer [2]. While smoking has been proven to have a high correlation with epigenetic changes in the DNA [3], other behavioral and environmental factors might also be recorded by changes in the epigenetics of the DNA (i.e. passive smoking, air pollution, occupational exposure, alcohol consumption, poor diet, low physical activity).

Adenocarcinoma (ADC) and squamous cell carcinoma (SCC) are the most common histological subtypes among all lung cancers. Both of them are a form of cancer that develops in the epithelial cells (carcinoma), and belong to the category of non-small cell lung cancer. Lung ADC develops in the glands that secrete products into the bloodstream or some other cavity in the body –the mucus secreting glands in the lungs. Most lung ADC arise in the outer, or peripheral, areas of the lung [4]. In contrast, lung SCC develops in flat surface covering cells. Squamous cells allow trans-membrane movement, like filtration and diffusion, for example the exchange of air in the alveoli of lungs. Squamous cells can also serve as boundary and protection of various organs. Most lung squamous cell cancers frequently arise in the central chest area in the bronchi [5].

The diagnosis of early stage lung cancer involves the use of imaging techniques, followed by a biopsy for pathology analysis [6]. Initially, screening of lung cancer is done using chest x-ray, or low-dose computed tomography. The American Cancer Society recommends screening to patients between the ages of 55–74 years old who are smokers or who quit smoking within the past 15 years [7]. Imaging techniques are not foolproof, so further analyses are usually required to make final diagnostic decisions. For instance, a cytological analysis is still required to confirm the imaging analysis [8]. In addition, tissue samples, albeit small, are often obtained during a needle aspiration biopsy or a bronchoscopy biopsy. The lack of tissue architecture in these small tissue specimens limits the pathologic analysis under a microscope [9].

Several studies have shown that molecular profiling of lung carcinoma is a viable tool for disease diagnosis [10], and prognosis [11]. What is more, distinguishing between ADC and SCC has significant clinical implications – both can have different treatment regimens. In this era of precision medicine, molecular characterization can be crucially important in the selection of an effective drug regimen. Potentially, patients can be subjected to drug regimens that are beneficial and/or harmful. Four possibilities summarize this situation: when a drug 1) has both therapeutic and adverse effects, 2) has only therapeutic effects (no adverse effects), 3) has adverse but no therapeutic effects, and 4) has no adverse nor therapeutic effects. Treatment safety and efficacy outcomes are important reasons of concern and the main reason for tumor subtyping [12]. Furthermore, ADC and SCC have distinct progression rate and progression free survival, which determines the selection of treatment [13].

The molecular mechanisms of ADC and SCC are considerably different. The standard molecular testing for lung cancer is to check for mutations of two molecules: epidermal growth factor receptor (EGFR) and rearrangement of anaplastic lymphoma kinase (ALK). Each protein has mutations that lead to the development of lung cancer. However, EGFR is found to be mutated only in around 10 % of tumors [14]. Similarly, ALK mutation occurs only in 6 % of tumors [15]. Although some drugs target EGFR and ALK positive tumors with therapeutic benefits for the patient, 75 % of lung tumors do not possess these molecular alterations [16]. The high sensitivity and low specificity of these diagnostic molecules is a motivation to research into new diagnostic models.

DNA methylation is an emerging diagnostic technology to measure the epigenetic changes in the DNA, characterized by the addition of a methyl group in regions of the DNA known by having CpG islands. Traditionally, gene expression has been used as a prognostic biomarker for lung carcinoma, and differentially expressed genes between lung cancer subtypes have been found [17]. However, it has been suggested that DNA methylation signatures of cancer should also be considered as a potential diagnostic biomarker of the disease [18]. Distinct DNA methylation signatures exist between ADC and SCC [19], and also between tumor tissue and normal surrounding tissue [20]. Since DNA methylation plays a significant role in the regulation of gene expression [21], there is an added value of investigating both data types.

Computational modeling methods, such as Bayesian classifiers, have been used successfully to model the complexity of genomic data. A study by Chang and Ramoni [22], yielded very high classification performance (accuracy = 0.95) to distinguish between lung tumor ADC and lung tumor SCC. Despite these results, the study still has open questions that are significant for the cause of precision medicine. For instance, selecting appropriate tissue samples to maximize microarray analysis is a big challenge. Inadequate biopsies can cause misdiagnosis and delay appropriate treatment [23]. In some cases, the amount of tissue available in the biopsy might not be enough to make a diagnosis from pathology and characterize the DNA changes in the cancer cells.

A major challenge of our study is the lack of tissue availability in public datasets. Typically, a biopsy tissue represents a very small portion of the lung. In spite of ultrasound guidance, it is easy to miss a small focal malignancy, and end up retrieving tumor-adjacent histologically-normal tissue (TAHN) along with Tumor tissue. In those cases, the biopsy is discarded if it cannot retrieve more than 50 % of tumor tissue [9]. The patient would have to undergo a new procedure to obtain another biopsy. Thus, it is worth exploring computational alternatives for classifying lung cancer subtypes given a small biopsy sample and a mix of TAHN and tumor tissue.

Our goal in this work was to test whether computational modeling can be a viable approach to accurately differentiate between lung cancer subtypes, given molecular profiles of tumor tissue using DNA methylation data. Specifically, we tested the hypothesis that “Bayesian modeling is sufficient to classify lung cancer subtypes, regardless of the tissue sample being tumor or tumor-adjacent.” In this paper, we evaluated the ability of a Bayesian classifier to accurately differentiate lung cancer subtypes using real lung cancer molecular profiling data sets that are also publicly available.

## Methods

### Datasets

To test our hypothesis, we extracted datasets containing gene expression and DNA methylation beta values from the Cancer Genome Atlas (TCGA) data portal for lung adenocarcinoma (LUAD [24]) and lung squamous cell carcinoma (LUSC, [25]). Additionally, we also used the gene expression dataset of lung adenocarcinoma patients, described by Landi et al. [26], GEO accession number GDS3257. Table 1 describes the characteristics of the samples we used for this study. For each dataset, it provides information on the type of ‘omic’ data type, source of data, assay platform, including number of features (i.e. genes or DNA methylation sites), and the number of sample distribution – that is, tumor tissue (T and TAHN) – within each subtype, where available. The formatted TCGA dataset used in this study, along with sample IDs, are provided in Additional file 1 (TAHN_ADC_ vs. Tumor_ADC_ in gene expression), Additional file 2 (TAHN_SCC_ vs. Tumor_SCC_ in gene expression), and Additional file 3 (TAHN_ADC_ vs. Tumor_ADC_ in methylation). The annotations from TCGA to identify these samples are provided in Additional file 4 (Appendix A).

**Table 1:** Datasets and sample distributions

Dataset Source	Tissue type	ADC	SCC
GEO: GDS3257 (gene expression)	Tumor	58	***
	TAHN	49	***
TCGA: LUAD+LUSC (gene expression)	Tumor	32	153
	TAHN	***	***
TCGA: LUAD+LUSC (DNA methylation)	Tumor	65	132
	TAHN	24	27

See challenge in Background on lack of TAHN tissue availability (***). GEO gene expression platform: Affymetrix Human Genome U133A Array (22,283 features), TCGA gene expression platform: Agilent 244 K Custom Gene Expression (17,814 features). TCGA methylation platform: Illumina Infinium HumanMethylation 27 k (27,578 features)

### Experimental design

**Fig. 1:**
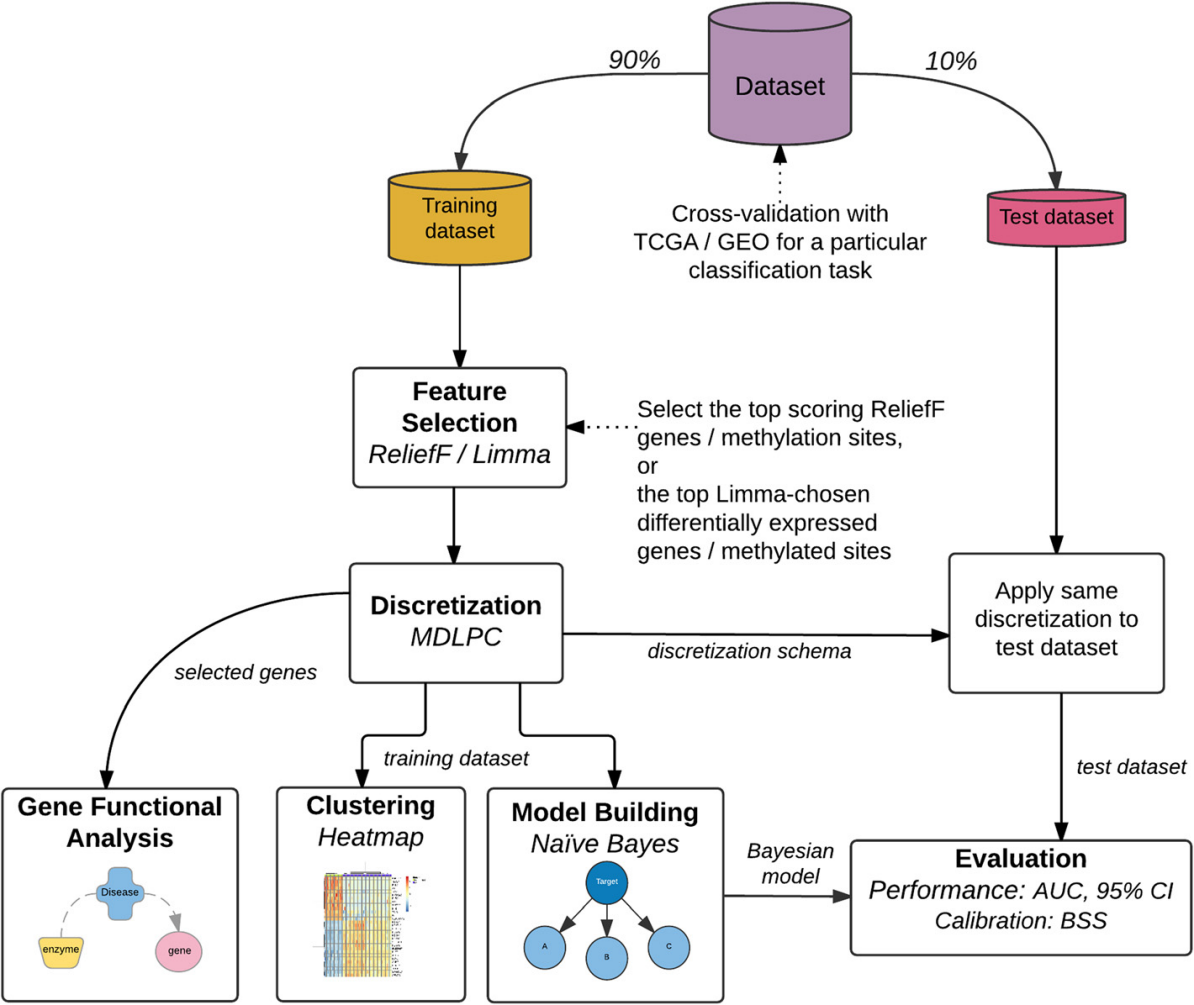
Cross-validation (10-folds) experimental design for a particular classification task, using feature selection and discretization. There are three outcomes: a simple naïve Bayesian model with its test evaluation; clustering of samples based on selected genes; and gene enrichment analysis. Algorithms: ReliefF, Limma, minimum description length principle cut (MDLPC). Evaluation: area under the receiver operating characteristic (AUC), 95 % confidence interval (CI), and Brier Skill Score (BSS)

We followed a supervised classification process on 10-fold cross-validation. That is, for each fold we partitioned the dataset into training and test, where the former contains 90 % of the samples, while the latter contains the remaining 10 %. We ensured that each partition maintains the same class distribution as the whole dataset (stratified). In each fold, we analyzed the datasets using the experimental design as illustrated in Fig. 1. According to the design, there are four main components, namely, a) Feature Selection, b) Discretization, c) Model Building and d) Evaluation. We additionally perform Gene Functional Analysis, and apply Clustering methods to better understand the characteristics of the features chosen by this framework. Below, we explain each component in detail.

### Feature selection

High-throughput platforms, such as gene expression and methylation microarrays, generate high-dimensional data that is typically very complex for analysis. Feature selection is a machine learning pre-processing step that tries to find a subset of the original variables (also called features or attributes) that are highly associated with the target class variable (i.e. phenotype, like a disease state). We used the ReliefF algorithm [27] to rank all variables and select the top scoring ones. ReliefF is a multivariate filter algorithm that estimates how well a given variable can distinguish the target class given the instances that are near to each other. The initial number of variables (17,814 in gene expression, and 27,578 in methylation) is reduced to the top 30 scoring variables. In previous studies [28], it has been reported that 30 is a sufficient number of genes to create computational classification models. With this number of genes, the classification models created would have a good trade-off between relevance and complexity of the model.

Similarly, we also selected the differentially expressed (DE) genes and differentially methylated (DM) probe sites from each dataset using Limma, which is an R-language package for the analysis of microarray data [29]. Limma uses a t-statistic to rank genes in order of evidence for differential expression. It first fits linear models for each gene (lmFit), and then it uses empirical Bayes (eBayes) moderation to adjust the standard error of the models by borrowing information from the rest of the genes (average variance across all genes). This method is very effective in finding differentially expressed (DE) genes in microarray data, however with methylation datasets it has not been equally successful [30]. The output of finding the DE genes and DM probe sites with *Limma* can be seen as a feature selection method (or ranked list). Similarly to the ReliefF selection, we selected the top 30 most DE genes and DM probe sites (based on log_2_-fold change) to build a classifier for comparison with ReliefF. The output of the resulting classifiers was evaluated using the area under the receiver operating characteristic curve (AUC) performance metric in the test datasets.

### Discretization

Most ‘omic’ data such as gene expression and methylation are represented with continuous values. However, many machine learning algorithms are designed to only handle discrete (categorical) data, using nominal variables, while real-world applications, like ‘omic’ data analysis, typically involves continuous-valued variables. Discretization, the process of transforming continuous values into discrete ones, has been shown to improve the performance of machine learning classifiers [31]. To discretize the variables, we used the Fayyad and Irani’s minimum description length principle cut (MDLPC) [32]. This method, which is widely used in the machine learning community, applies a supervised greedy search strategy to recursively find the minimal number of cut-points in each variable that minimizes the entropy of the resulting subintervals.

For continuous methylation values ranging from 0 to 1, three possible strategies for discretization can occur. The first strategy is when a fixed cut-point is determined arbitrarily for all variables (for example, choosing > 0.5 methylated, while ≤ 0.5 could refer to unmethylated). The second strategy, when an expert-based discretization is made for all variables (i.e. unmethylated < 0.1, partially methylated between 0.1 and 0.8, and methylated > 0.8 [33]). The third strategy is when a supervised discretization method creates independent cut-points for each variable. For the first and second strategies, the same discretization scheme (i.e. same number of intervals or cut-points) is used for all variables. However, this approach is suboptimal for a classification task. For instance, when using MDLPC we observed that the methylation site cg19782598 was discretized into two categories: methylated (>0.86) and unmethylated (≤0.86); while methylation site cg11693019 was discretized into three categories: methylated (>0.76), partially methylated (between 0.76 and 0.47), and unmethylated (<0.47). Thus, supervised discretization could help identify appropriate cut-points for each variable, as opposed to the others, which naïvely assume the same cut-points for variables.

### Clustering

In computational genomics, heatmaps are used to graphically show the level of expression that a selected group of genes have in a cohort of patient samples. A heatmap can also be built with methylation intensity values. We build heatmaps from the genes selected by Limma and ReliefF to further validate the results obtained with these feature selection methods. The clusters are a visual representation of the class discrimination ability of the genes selected.

The order in which genes (rows) and samples (columns) are ordered in the heatmap matrix is often based on an agglomerative hierarchical clustering. We used the Minkowski measure to calculate the pairwise distances between elements, and then aggregated the closest elements in clusters using the Ward linkage calculation of distances between clusters. This combination of Minkowski distance and Ward linkage has been shown to perform well in biomedical and synthetic datasets [34].

### Gene functional analysis

We also performed Gene Functional Analysis using QIAGEN’s Ingenuity® Pathway Analysis tool (IPA®, QIAGEN Redwood City, www.ingenuity.com) to gain insight into the biological role of the genes selected by our framework. First, all gene symbols selected were used as input for the IPA platform, which will search for correlations between these genes and functions or pathways in their curated literature. A p-value is computed using Fisher’s right-tailed exact test for the gene list to a function/pathway it may be associated with. The p-values indicate the likelihood of association between the gene set (as selected by ReliefF) and a specific function (set of genes associated with a function) to have occurred due to random chance alone. A p-value of less than 0.05 is considered to be significantly better than random chance. Methylation probe sites were mapped into their corresponding gene symbols that they methylate.

### Model building

In the machine learning literature, a classifier is a computational model that can differentiate between two (or more) states of disease. Bayesian networks [35] are particularly useful classifiers that are very popular in the classification of biomedical data. A Bayesian network (BN) is a probabilistic graphical representation of random variables (nodes) and probabilistic dependencies among them (arcs). Once a Bayesian network is learned, the structure and conditional probability tables can be used to calculate the posterior probabilities for a new case to be a member of a given class, i.e. the probabilities of a new case being ADC given the BN and the data. P(ADC = True|BN, data). A special case of BN is the naïve Bayesian classifier (NB), which assumes a strong conditional independence among the variables. In a NB structure, the target node (i.e. class variable) is the parent for all other features, and there are no arcs among those children nodes. The child nodes are independent given the parent, which facilitates the calculation of posterior probabilities by substituting the joint probability with the product of their probabilities. NBs have been shown to predict poorly in high-dimensional genomic datasets [36], but it is expected that the use of a feature selection method (ReliefF or Limma) will improve the NB classification performance. Moreover, its simplicity makes it a powerful tool to be considered in a biomedical classification framework, while giving us insights into the baseline performance on a given dataset.

### Evaluation

We evaluated the NB classifiers using the area under the receiver operating characteristic (AUC), which is a measurement of the area created by plotting the performance of a classifier for the true positive rate versus the false positive rate. When presented with a test dataset, the Bayesian network calculates a posterior probability for every case, and a threshold is used to assign the class for the new cases. The curve is constructed by varying the threshold to which the probability is considered for class determination. Also, the 95 % confidence interval (C.I.) of the AUC was calculated using DeLong’s method for variance estimation [37].

AUC (equivalent to c-statistic) is a useful measurement of the ability of models to discriminate between two (or more) classes [38]. Calibration deals with agreement between observed outcomes and predictions. For this purpose, we used the Brier Skill Score (BSS) [39] creates an index between −1 and 1 that provides information as of how far away the results of any classifier are in relation to the unskilled classifier. The unskilled classifier is one that only considers the distribution of data. A classifier with a positive BSS would therefore be skilled and unbiased.

## Results

We investigated four classification tasks depending on the tissue type. These tasks test our hypothesis that the TAHN tissue has distinct genomic signatures that can differentiate among non-small cell lung cancer subtypes. We describe the classification tasks as follows:

1. *TAHN*_*ADC*_ vs. *Tumor*_*ADC*_, and TAHN_SCC_ vs Tumor_SCC_, searches for molecular differences between tumor tissue and TAHN tissue. These tasks are only applied to one lung cancer subtype at a time, either adenocarcinoma or squamous cell carcinoma patients;
2. *Tumor*_*ADC*_ vs. *Tumor*_*SCC*_, which searches for molecular differences between subtypes using only Tumor tissue;
3. *TAHN*_*ADC*_ vs. *TAHN*_*SCC*_, which searches for molecular differences between subtypes using only TAHN tissue; and
4. *TAHN-Tumor*_*ADC*_ vs. *TAHN-Tumor*_*SCC*_, which searches for molecular differences between subtypes using both TAHN and Tumor tissue.

The classification performance for every naïve Bayes classifier was calculated by averaging the AUCs over all folds from the experimental design illustrated in Fig. 1. Table 2 shows results for the classification tasks, including 95 % confidence interval (C.I.) and Brier Skill Score (BSS) as a calibration measurement. Contingency tables for these models can be seen in Additional file 4 (Appendix B).

**Table 2:** AUC classification performance for different classification tasks

Classification Task	Omic	Feature selection with ReliefF			Feature selection with Limma		
		AUC	95 % C.I.	BSS	AUC	95 % C.I.	BSS
TAHN_ADC_ vs. Tumor_ADC_	G	0.99	0.97–1.0	0.89	0.94	0.82–1.0	0.73
	M	1.0	1.0–1.0	0.99	0.81	0.58–0.97	0.17
TAHN_SCC_ vs. Tumor_SCC_	M	1.0	0.99–1.0	0.94	0.99	0.96–1.0	0.66
Tumor_ADC_ vs. Tumor_SCC_	G	0.89	0.83–0.96	0.29	0.90	0.89–0.9	0.81
	M	0.97	0.94–0.99	0.71	0.89	0.74–1.0	0.38
TAHN_ADC_ vs. TAHN_SCC_	M	1.0	1.0–1.0	0.92	1.0	1.0–1.0	0.99
TAHN-Tumor_ADC_ vs. TAHN-Tumor_SCC_	M	0.92	0.89–0.95	0.42	0.94	0.87–1.0	0.56

G: gene expression, M: DNA methylation. The Brier Skill Score is a measurement of calibration of the classifier. A positive value on the BSS means that the classifier is well calibrated. A baseline classification is the work by Chang and Ramoni [22] which obtained an accuracy of 0.95 in the classification task Tumor_ADC_ vs. Tumor_SCC_

**Fig. 2:**
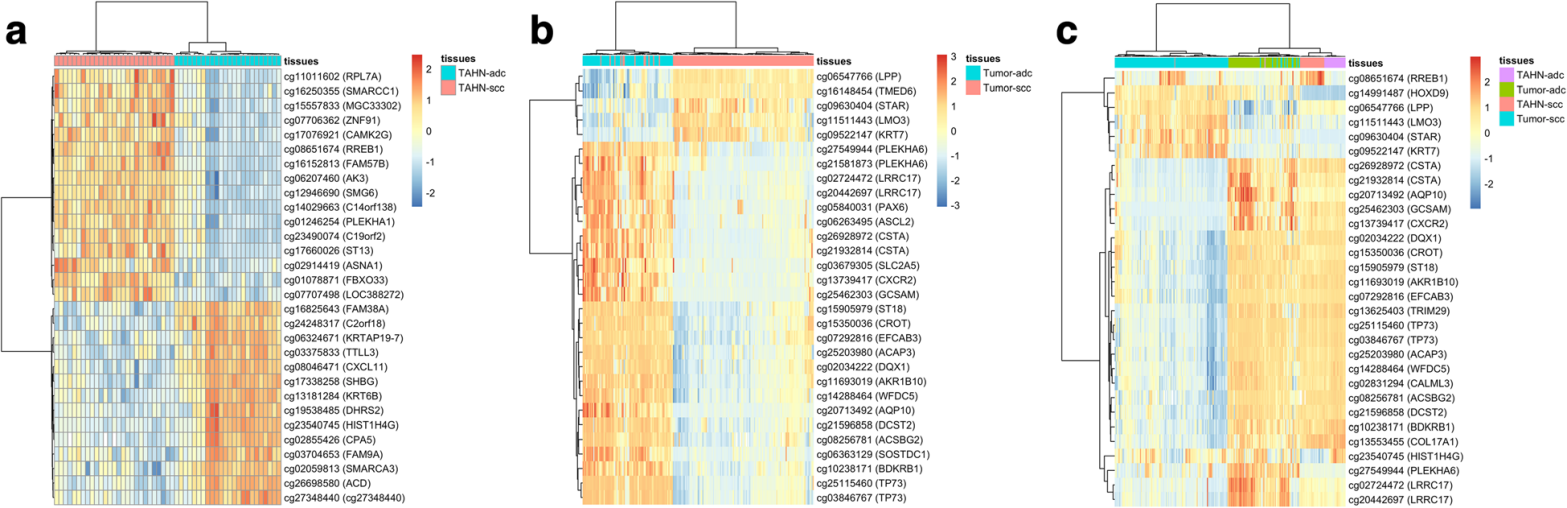
Heatmaps for classification task **a** TAHN_ADC_ vs. TAHN_SCC_, **b** Tumor_ADC_ vs. Tumor_SCC_ and **c** TAHN-Tumor_ADC_ vs. TAHN-Tumor_SCC_ using the ReliefF feature selection algorithm. In the vertical axis the corresponding methylation site and gene symbol (in parenthesis) are shown. Some methylation sites do not lie in a particular gene, therefore, no symbol is provided. When multiple methylation sites are selected for the same gene, these sites should have similar methylation intensity, for it to be included. In the horizontal axis, a color-coded representation of the tissue samples is provided. Two distinct groups are observed in all three heatmaps. Cluster purity (accuracy by classification using clustering) for each task is calculated to be 1.0, 0.94 and 0.85 respectively

All classification tasks achieved high predictive performances with AUC values higher than 0.8. For these datasets, the classification performance was similar between the NB classifiers created after applying ReliefF and Limma as feature selection methods. Limma is a popular method, among the genomics community, for the selection of differentially expressed genes, but it is not used as a feature selection method by the machine learning community. In contrast, ReliefF is a popular method among machine learning studies but not widely used in genomic studies. Figure 2 shows heatmaps and clusters for each classification task with the methylation probe sites selected using ReliefF.

**Fig. 3:**
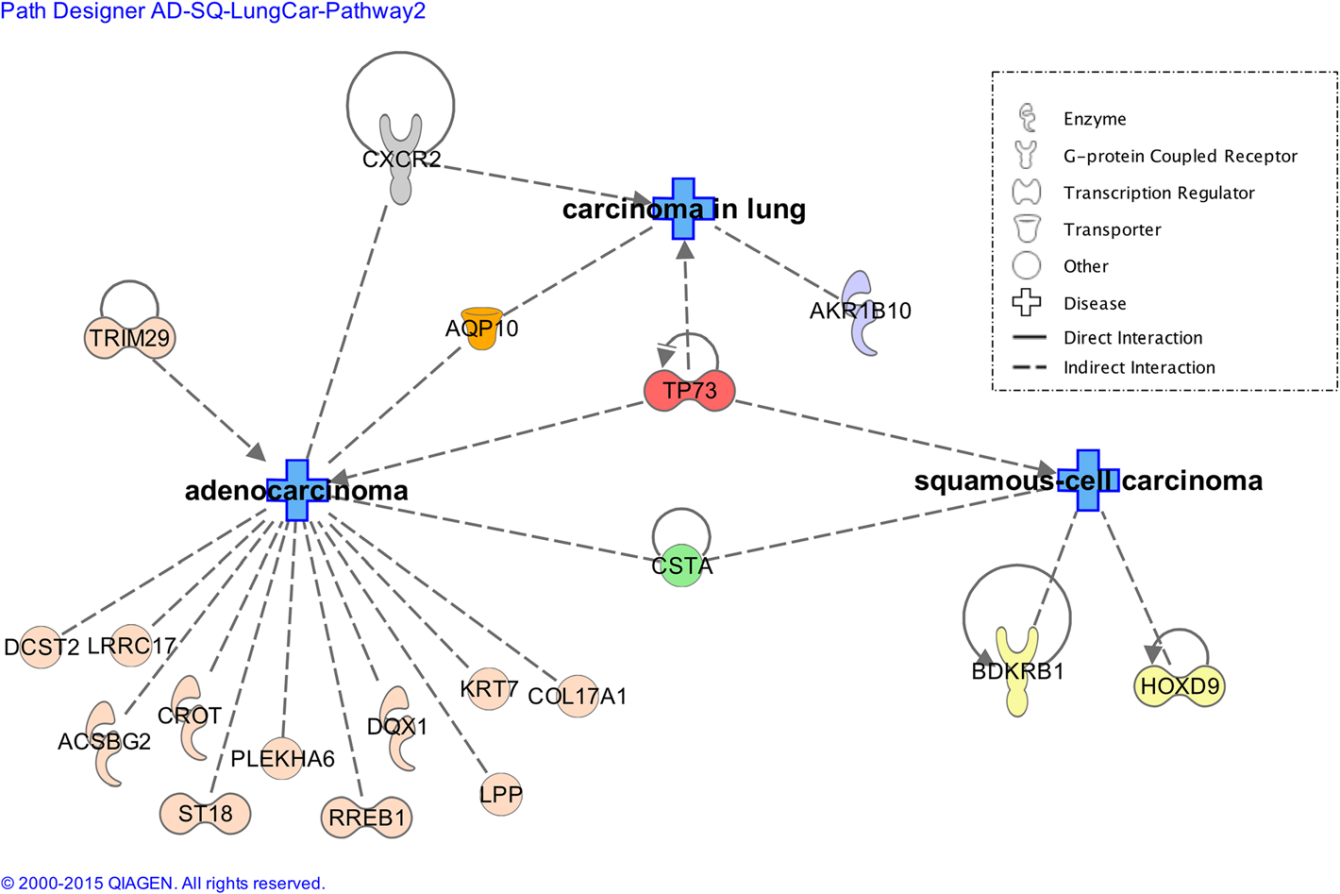
Gene interaction network generated by the IPA® software. It shows an analysis of the genes found by ReliefF in the classification task TAHN-Tumor_ADC_ vs TAHN-Tumor_SCC_. Three diseases are being shown (carcinoma of the lung, adenocarcinoma and squamous cell carcinoma), and the selected genes from our analysis were connected to these diseases via literature evidence that indicates: direct interactions (straight line), or indirect interactions (dashed line). Some of those interactions have arrow-heads indicating causation (e.g. BDKRB1). An arrow-head with a bar (i.e., TP73) indicates inhibition

We analyzed the genes found by ReliefF in the classification task of TAHN-Tumor_ADC_ vs TAHN-Tumor_SCC_ using IPA®. The results of the IPA® core analysis show a significant association between ReliefF-selected genes and the following diseases: cancer (25 out of 27) connective tissue disorder (13 out of 27), dermatological diseases and conditions (13 out of 27). Interestingly, the ReliefF-selected genes (19 out of 27) are associated with either adenocarcinoma (16 genes), squamous-cell carcinoma (4 genes) or carcinoma of the lung (4 genes). The list of genes and their associations can be seen in Table 3. Using these interesting 19 genes, we generated a gene interaction network to graphically visualize the relationships between genes and the disease class (adenocarcinoma, squamous-cell carcinoma and carcinoma of the lung). The network is illustrated in Fig. 3.

**Table 3:** Genes selected for the classification task of TAHN-Tumor_ADC_ Vs. TAHN-Tumor_SCC_

Gene Symbol	Gene Name	Known Literature Evidence to Cancer
ST18	suppression of tumorigenicity 18, zinc finger	Yes [45]
CSTA	cystatin A (stefin A)	Yes [45, 46]
LPP	LIM domain containing preferred translocation partner in lipoma	Yes [45]
CROT	carnitine O-octanoyltransferase	Yes [45]
BDKRB1	bradykinin receptor B1	Yes [47]
AKR1B10	aldo-keto reductase family 1, member B10 (aldose reductase)	Yes [48]
TP73	tumor protein p73	Yes [49–51]
EFCAB3	EF-hand calcium binding domain 3	Yes
RREB1	ras responsive element binding protein 1	Yes [45]
HIST1H4G	histone cluster 1, H4g	No
STAR	steroidogenic acute regulatory protein	Yes
ACSBG2	acyl-CoA synthetase bubblegum family member 2	Yes [45]
DQX1	DEAQ box RNA-dependent ATPase 1	Yes [45]
AQP10	aquaporin 10	Yes [45]
PLEKHA6	pleckstrin homology domain containing, family A member 6	Yes [52, 53]
GCSAM	germinal center-associated, signaling and motility	No
WFDC5	WAP four-disulfide core domain 5	Yes
KRT7	keratin 7, type II	Yes [54]
DCST2	DC-STAMP domain containing 2	Yes [45]
CALML3	calmodulin-like 3	Yes
ACAP3	ArfGAP with coiled-coil, ankyrin repeat and PH domains 3	Yes
LRRC17	leucine rich repeat containing 17	Yes [45]
TRIM29	tripartite motif containing 29	Yes [55]
CXCR2	chemokine (C-X-C motif) receptor 2	Yes [45, 56, 57]
HOXD9	homeobox D9	Yes [58]
COL17A1	collagen, type XVII, alpha 1	Yes [45]
LMO3	LIM domain only 3 (rhombotin-like 2)	Yes

The list of genes is ordered by their ranks, as selected by ReliefF for the classification task of TAHN-Tumor_ADC_ Vs. TAHN-Tumor_SCC_. The Entrez gene symbol, and the gene name are listed in the first two columns respectively. The ‘Known Literature Evidence to Cancer’ indicates if links to cancer were detected by the IPA® software. Citations are provided to literature indicating links to adenocarcinoma, squamous-cell carcinoma and carcinoma in lung

## Discussion

### Evaluation of classifiers

The classification performance for all models is high ($\mathrm{A}\mathrm{U}\mathrm{C}\ge 0.81$), with positive calibration ($\mathrm{B}\mathrm{S}\mathrm{S}>0$). This positive calibration is a good indication that the models will perform well for other cases, and that they were not biased by the distribution of the data.

In the classification task of TAHN_ADC_ vs. Tumor_ADC_, the naïve Bayesian model created obtained high predictive performances ($\mathrm{A}\mathrm{U}\mathrm{C}\ge 0.99\;\mathrm{with}\;\mathrm{ReliefF},\;\mathrm{and}\ge 0.81\;\mathrm{with}\;\mathrm{Limma}$). The classification task TAHN_SCC_ vs. Tumor_SCC_ also obtained high predictive performances ($\ge 0.99\;\mathrm{with}\;\mathrm{both}\;\mathrm{feature}\;\mathrm{selection}\;\mathrm{methods}$). The molecular differences between TAHN and tumor tissue show distinctive signatures regardless of ‘omic’ dataset, feature selection method or lung cancer subtype. The results for these classification tasks were as expected since the tissue architecture between TAHN and Tumor is recognizable under a microscope if enough tissue samples are provided. They also could be achieved with the relatively small number of normal tissues available for analysis, since these normal tissues are very homogenous in expression and methylation features.

In the classification task of Tumor_ADC_ vs. Tumor_SCC_ the predictive performance was very high ($\mathrm{A}\mathrm{U}\mathrm{C}\ge 0.89,\;\mathrm{f}\mathrm{o}\mathrm{r}\;\mathrm{gene}\;\mathrm{expression},\;\mathrm{and}\ge 0.89\;\mathrm{with}\;\mathrm{methylation}$). Previous studies for the same classification task also show a similar classification performance. For example, Ben-Hamo et al. [40] correctly classified 85 %, using linear models. Meanwhile, Cai et al. [10] obtained an accuracy of 86 % using ensemble methods; Li et al. [41] achieved an AUC of 0.98 using Support Vector Machines; and Zhang et al. [42] achieved AUCs of 0.89 using naïve Bayesian models. Similarly, the study by Chang and Ramoni [22] achieved an accuracy of 0.95, using naïve Bayesian models. It is worth noting that none of these studies used methylation datasets and they fail to clearly recognize the importance of TAHN tissue for classification.

The classification task of TAHN_ADC_ vs. TAHN_SCC_ also had very high evaluation performances ($\mathrm{A}\mathrm{U}\mathrm{C}=1$). This high performance means that all samples were correctly classified. We hypothesize that an explanation of this excellent result can be attributed to the distinctive epigenetic differences between lung tissues. We did not evaluate the gene expression in this classification task due to the lack of an available dataset. To the best of our knowledge reporting of TAHN tissue in public repositories is still an open challenge that should be addressed to improve experimental designs of other studies. A study by Haaland et al. [43], showed that there are differentially expressed genes between TAHN tissues in prostate cancer. In our study, we investigate DNA methylation data to indicate that the same differences could also be found in lung cancer TAHN tissues, and we hypothesize that the use of TAHN tissues might also help in the classification performance of other cancer types.

The classification task of TAHN-Tumor_ADC_ vs. TAHN-Tumor_SCC_ is a novel approach, where a mix of tissue types are used to classify between lung cancer subtypes. The noise introduced by mixing tissue types is overcome by our experimental design, which was able to obtain a very good classification performance ($\mathrm{A}\mathrm{U}\mathrm{C}\ge 0.92$). Despite, the ‘noisy’ tissue samples, a simple naïve Bayesian classifier can accurately classify between lung cancer subtypes. This classification performance is confirmed by the heatmap analysis in Fig. 2c, where the tumor tissue of ADC creates a distinct cluster, while the remaining samples cluster together in three distinct subclusters. Furthermore, our Gene Functional Analysis using IPA® shows strong associations to cancer pathways, with 19 genes found to be associated with adenocarcinoma, squamous-cell carcinoma and carcinoma of the lung. Out of these 19 genes we found 4 genes associated specifically with lung cancer subtypes: AKR1B10, AQP10, CXCR2, TP73.

### The value of using TAHN tissue for classification

Lung cancer patients could benefit with a potentially novel approach for subtyping. The diagnosis of adenocarcinoma vs. squamous cell carcinoma is routinely accomplished using histology supplemented by immunohistochemistry (TTF-1 and p63/p40). It is therefore not likely that our approach would change this practice, which is well established, quick and inexpensive. Rather, we suggest that the use of epigenomic changes could help in the small number of tumors which remain difficult to classify. However, the primary importance of our work may be in providing additional understanding of the origins of squamous cell and adenocarcinomas, which suggest that these phenotypes are associated with, or perhaps even derived from, different epigenomic phenotypes. Epigenomic alterations, in the form of DNA methylation, prevent the binding of transcription machinery, resulting in gene silencing [44]. Moreover, DNA methylation signatures are different between tissue types and between tumors and normal surrounding tissue [20]. In our study, tumor-adjacent histologically normal tissue samples were used to classify lung cancer subtypes with excellent results. This classification performance was achieved when no tumor samples were involved (TAHN_ADC_ vs. TAHN_SCC_), and when a mix of tissue was used (TAHN-Tumor_ADC_ vs. TAHN-Tumor_SCC_). The high AUC results are an indication of the diagnostic potential of this technology.

### Limitations and future work

Our study had some limitations, which include the following: 1) There were a limited number of tumor-adjacent histologically normal tissue samples used. However, the homogeneity of these normal tissues we observed suggests that additional normal tissues would not improve the classifier. 2) The resulting classifiers were not validated in another dataset outside of TCGA lung samples. 3) Each ‘omic’ classifier is independent of one another. In the future, we would like to explore data integration models in a multi-omic approach. 4) The classification problem of discriminating cancer subtypes of adenocarcinoma and squamous cell carcinoma could also be explored in a pan-cancer analysis, to validate the same finding seen in our study of lung cancer subtypes. 5) Due to the challenge of data availability, in this study we did not analyze biopsies with varying percentages of tumor and TAHN tissue (mixed biopsies). Instead, we took relatively ‘pure’ biopsies of either tumor or TAHN to classify between lung cancer subtypes. A future study could consider the molecular classification or discovery of cancer given a mixture of tumor and TAHN tissue. For example, an analysis of ‘omic’ data from cancerous and non-cancerous tumor tissues, as well as TAHN tissue for both types of tumors, might be performed in the same way as presented in this manuscript.

## Conclusions

In this paper, we addressed the issue of lung cancer subtyping using DNA methylation data from TAHN tissue, which is a novel strategy for classification of non-small cell lung cancer samples. This study demonstrated that using computational Bayesian modeling, it is possible to discover the molecular differences between tumor and tumor-adjacent tissue of lung cancer patients. This discovery will allow clinicians to use the available biopsy material without worrying about its tissue composition, yielding in less invasive diagnostic procedures for the patient. We hope that our results will encourage researchers to also make use of TAHN tissue samples generated in their laboratories for predictive modeling and make this data available for public use. As more data becomes available, our models can be further improved, and future discoveries could be made in other cancers.

## Availability of supporting data

The datasets used in this study are publicly available from The Cancer Genome Atlas (https://tcga-data.nci.nih.gov/tcga/) in datasets LUAD and LUSC; and also from the Gene Expression Omnibus (http://www.ncbi.nlm.nih.gov/geo/), accession number GDS3257. The formatted datasets used in this study, along with sample IDs, are provided in Additional file 1 (TAHN_ADC_ vs. Tumor_ADC_ in gene expression), Additional file 2 (TAHN_SCC_ vs. Tumor_SCC_ in gene expression), and Additional file 3 (TAHN_ADC_ vs. Tumor_ADC_ in methylation). The annotations from TCGA to identify these samples are provided in Additional file 4 (Appendix A).

## Additional files

Additional file 1: Formatted TCGA dataset used in this study, along with sample IDs for classification task TAHN_ADC_ vs.Tumor_ADC_ in gene expression. (CSV 5182 kb) Additional file 2: Formatted TCGA dataset used in this study, along with sample IDs for classification task TAHN_SCC_ vs.Tumor_SCC_ in gene expression. (CSV 24086 kb) Additional file 3: Formatted TCGA dataset used in this study, along with sample IDs for classification task TAHN_ADC_ vs.Tumor_ADC_ in DNA methylation. (CSV 41150 kb) Additional file 4: Appendix A shows the Cancer Genome Atlas annotations to identify the types of samples used in this study.Appendix B shows additional performance measures for the models described. (DOCX 106 kb)
