# Supplementary material for: On Predicting lung cancer subtypes using ‘omic’ data from tumor and tumor-adjacent histologically-normal tissue
Source: BMC Cancer. 2016 Mar 4;16:184. doi: 10.1186/s12885-016-2223-3 (PMC4778315; doi:10.1186/s12885-016-2223-3)
Supplement: Additional file 4: — Appendix A shows the Cancer Genome Atlas annotations to identify the types of samples used in this study.Appendix B shows additional performance measures for the models described. (DOCX 106 kb) [file 12885_2016_2223_MOESM4_ESM.docx]

**On Predicting Lung Cancer Subtypes using 'Omic' Data from Tumor and Tumor-Adjacent Histologically-Normal Tissue**

Arturo López Pineda^1, *^, Henry Ato Ogoe^1^, Jeya B. Balasubramanian^1^, Claudia Rangel Escareño^2^, Shyam Visweswaran^1^, James G. Herman^3^, Vanathi Gopalakrishnan^1^

^1^University of Pittsburgh School of Medicine. Department of Biomedical Informatics, 5607 Baum Boulevard, 15206 Pittsburgh, PA, USA.

^2^National Institute of Genomic Medicine. Department of Computational Genomics, Periferico Sur No. 4809, Col. Arenal Tepepan, Tlalpan, 14610 Mexico City, Mexico.

^3^University of Pittsburgh School of Medicine. Department of Medicine. Division of Hematology/Oncology. UPMC Cancer Pavilion, 5150 Centre Avenue, 15232 Pittsburgh, PA, USA.

# Appendix A

This Appendix includes annotations of the subjects used in this study from The Cancer Genome Atlas (TCGA).

**Annotations**

| **Adenocarcinoma (ADC)** |
| --- |
| Lung Adenocarcinoma Mixed Subtype  Lung Adenocarcinoma- Not Otherwise Specified (NOS)  Lung Mucinous Adenocarcinoma  Lung Acinar Adenocarcinoma  Lung Bronchioloalveolar Carcinoma Mucinous  Lung Papillary Adenocarcinoma  Lung Micropapillary Adenocarcinoma  Mucinous (Colloid) Carcinoma  Lung Clear Cell Adenocarcinoma  Lung Bronchioloalveolar Carcinoma Nonmucinous  Lung Solid Pattern Predominant Adenocarcinoma  Lung Signet Ring Adenocarcinoma" |

| **Squamous Cell Carcinoma (SCC)** |
| --- |
| Lung Squamous Cell Carcinoma- Not Otherwise Specified (NOS)  Lung Basaloid Squamous Cell Carcinoma  Lung Papillary Squamous Cell Carcinoma  Lung Small Cell Squamous Cell Carcinoma |

We used the Sample Type description from the TCGA to select the normals and controls.

https://tcga-data.nci.nih.gov/datareports/codeTablesReport.htm?codeTable=Sample%20type

| **Normal Samples** |
| --- |
| 10 Blood Derived Normal  11 Solid Tissue Normal  12 Buccal Cell Normal  13 EBV Inmortalized Normal  14 Bone Marrow Normal  20 Control Analyte  40 Recurrent Blood Derived Cancer – Peripheral Blood  50 Cell Lines  60 Primary Xenograft Tissue  61 Cell Line Derived Xenographt Tissue |

| **Tumor Samples** |
| --- |
| 01 Primary Solid Tumor  02 Recurrent Solid Tumor  03 Primary Blood Derived Caner – Peripheral Blood  04 Recurrent Blood Derived Cancer – Bone Marrow  05 Additional – New Primary  06 Metastatic  07 Additional Metastatic  08 Human Tumor Original Cells  09 Primary Blood Derived Cancer – Bone Marrow |

# Appendix B

This Appendix shows additional performance measures for the models described in the main manuscript. The threshold used is 0.5

| **Measurement** | **TAHN_ADC_ vs. TAHN_SCC_** | **TAHN-Tumor_ADC_**  **vs.**  **TAHN-Tumor_SCC_** | **Tumor_ADC_ vs. Tumor_SCC_** |
| --- | --- | --- | --- |
| **Threshold** | 0.5 | 0.5 | 0.5 |
| **Positive class** | TAHN_ADC_ | TAHN-Tumor_ADC_ | Tumor_ADC_ |
| **Negative class** | TAHN_SCC_ | TAHN-Tumor_SCC_ | Tumor_SCC_ |
| **Accuracy** | 98.04 | 85.08 | 92.89 |
| **AUC** | 1.00 | 0.92 | 0.97 |
| **Sensitivity** | 95.83 | 96.63 | 96.92 |
| **Specificity** | 100.00 | 78.62 | 90.91 |
| **PPV** | 100.00 | 71.67 | 84.00 |
| **NPV** | 96.43 | 97.66 | 98.36 |

The observed best thresholds for each classification task are provided below, as inferred by the ROC curve:

| **Measurement** | **TAHN_ADC_ vs. TAHN_SCC_** | **TAHN-Tumor_ADC_**  **vs.**  **TAHN-Tumor_SCC_** | **Tumor_ADC_ vs. Tumor_SCC_** |
| --- | --- | --- | --- |
| **Threshold** | 1E10^-6^ | 0.99 | 0.05 |
| **Accuracy** | 100.00 | 86.69 | 93.91 |
| **Sensitivity** | 100.00 | 96.63 | 100.00 |
| **Specificity** | 100.00 | 81.13 | 90.91 |
| **PPV** | 100.00 | 74.14 | 84.42 |
| **NPV** | 100.00 | 97.73 | 100.00 |
